# High cardiovascular disease mortality after penile squamous cell carcinomas diagnosis: Results from the United States SEER population, 2005-2016

**DOI:** 10.3389/fonc.2022.1004791

**Published:** 2022-10-14

**Authors:** Zequan Xie, Xiangpeng Zhan, Yunwei Zheng, Yang Liu, Tao Chen, Ming Jiang, Yu Li, Bin Fu

**Affiliations:** ^1^ Urology Department, The First Hospital Of Putian City, Putian, China; ^2^ Department of Urology, The First Affiliated Hospital of Nanchang University, Nanchang, China; ^3^ Department of Cardiology, The Second Affiliated Hospital of Nanchang University, Nanchang, China

**Keywords:** penile squamous cell carcinomas, cardiovascular disease, Surveillance, Epidemiology and End Results (SEER), cause of death, prognosis, mortality

## Abstract

**Background:**

Cancer survivorship care is an emerging and necessary component of oncology management. To explore cardiovascular disease (CVD)-specific mortality and prognostic factors among patients with penile squamous cell carcinomas (PSCC). These results aid clinicians in furtherly understand this disease’s prognosis.

**Method:**

We analyzed Surveillance, Epidemiology and End Results Program data for 2668 PSCC cases diagnosed between 2005 to 2016. We calculated standardized mortality ratios (SMRs) of CVD and all-cause mortality, comparing PSCC patients with general population men. A cumulative mortality curve and competitive risk regression model were utilized to evaluate the prognostic factors of CVD-specific death.

**Results:**

Death distribution is as follows: PSCC (42.4%), other causes (21.3%) CVD (19%), and other cancers (17.3%). PSCC patients are more like to die from CVD (SMR=3.2, 95%CI: 3.1-3.3) and all-cause death compared with the general population. Meanwhile, patients undergoing surgery show a relatively higher CVD-specific mortality than the general population (SMR=2.7, 95%CI: 2.4-3.2). In the competitive risk model, higher CVD mortality is associated with age, region, year of diagnosis, stage, and marital status (all P<0.05). Patients with the localized stage show a higher risk of CVD-specific death than those with regional or distant stage.

**Conclusion:**

Our study mainly reveals that cardiovascular disease was the important cause of death and higher CVD-specific mortality among PSCC patients. Several associated factors related to CVD-specific death are also identified. In the future, more work in educating health care professionals on the components of survivorship care is needed to meet the long-term and late effects cancer patients experience.

## Introduction

Penis cancer is a rare disease, accounting for 0.4% to 0.6% of all malignancies in men in the United States and Europe (1). About 95% penis cancer derives from the squamous cells of glandular and preputial skin and is diagnosed with penile squamous cell carcinomas (PSCC) (2). PSCC presented great regional heterogeneity. For example, the incidence rate is 0.1-1 per 100,000 men in high-income countries. At the same time, it accounts for 10% of the total number of male malignancies in some African, Asia, and South American countries. The primary reason for this heterogeneity may be related to confirmed risk factors, including circumcision practices, infection with human papillomavirus (HPV), smoking, obesity, chronic inflammation, and so on (3–5).

PSCC patients diagnosed with the localized stage (about 40%) showed relatively good survival outcomes and reported data suggested that the overall survival rate of these patients was as high as 90% (5). However, once the tumor metastases, the prognosis will deteriorate sharply. The management of PSCC has always been a comprehensive and complex challenge, considering that cancer has a far-reaching physiological and psychological impact on the quality of life of patients and survivors by altering sexual and urinary functions (2). There is increasing evidence that the management of co-morbidities, especially cardiovascular disease (CVD), should receive more attention, considering that the survival of penile squamous cell carcinomas has not significantly improved since 1990 in the United States (6). The main reason for this consideration is that cardiovascular disease has become the leading cause of death for cancer patients in recent years (7, 8). Meanwhile, patients with PSCC are diagnosed at a relatively high age (range 50 to70 years), the age group with a high incidence of cardiovascular disease (1). Moreover, effective lifestyle interventions and other strategies to prevent CVD complications exist but may not be fully utilized in this population (9, 10). Therefore, strengthening the understanding and management of CVD may effectively improve survival in patients with PSCC.

Up to our knowledge, there is still a lack of studies focusing on CVD in PSCC patients considering the study population is relatively small. Therefore, we have explored the U.S. Surveillance, Epidemiology, and End Results Program (SEER) database to obtain patients with PSCC. The primary purpose of this study is to explore the distribution of CVD-specific death among PSCC patients stratified by characteristics. In addition, the risk factors of CVD-specific death were investigated by utilizing the Fine-Gray multivariable-adjusted competing risks model.

## Methods

### Study population and variables description

The study involved a secondary analysis of the National Cancer Institute’s SEER project database. We identified PSCC patients who were histologically diagnosed with penile squamous cell carcinoma based on the list of ‘*Site Recode ICD-O-3/WHO 2008 classification*’ and ‘ *Histology recode - broad groupings*’ between 2005 and 2016 from the SEER 18 registries research database, covering approximately 28% of the U.S. population (based on the 2010 census). We excluded the following patients: age at diagnosis <15 years, unknown survival months, and unknown cause of death.

We used the SEER * stat (version 8.2.1) to generate a list of cases and incorporated the following variables: age at diagnosis, race (white, black, and other, unknown), year of diagnosis, marital status (married, separated, or divorced or widowed (SDW), single, unknown), PRCDA region (east, northern plains, pacific coast, southwest), SEER historic stage (Localized, Regional, Distant, unknown), surgery record, survival month, and cause of death (COD) to site recode. We divided the causes of death into four categories based on the International Classification of Diseases, tenth Edition (ICD-10): PSCC, cardiovascular diseases, other cancers, and other causes.

### Statistical analysis

We described the distribution of causes of death in PSCC patients by the index of the proportional mortality ratio (PMR), which was calculated by dividing the number of patients who died from special causes (PSCC, cardiovascular diseases, other cancers, and other causes) by the total number of PSCC patients. In addition, the study population was stratified by age, race, year of diagnosis, region, tumour stage, operation records, and the cause of death distribution in the subgroups were further examined. We compared CVD and all-cause mortality between PSCC patients in the SEER cohort and general men in the American population and expressed it as standardized mortality (SMR). The age-specific risks of CVD and all-cause death were obtained from the Centers for Disease Control and Prevention Wide-ranging Online Data for Epidemiologic Research (WONDER) Tool (11). The expected number of deaths in each age group was calculated by multiplying the number of PSCC patients in each age group by the age-specific crude risk in the general population. The SMRs were calculated according to the number of observed PSCC deaths divided by the expected number of deaths. If SMRs were more significant than one and the P-value< 0.05, it indicates that the cardiovascular mortality rate of patients with PSCC was higher than that of the general population in the United States, and the results were statistically significant. The Fine-Gray multivariable-adjusted competing risks model was performed to adjust confounding factors (including age, race, marital status, region, year of diagnosis, grade, and stage) and evaluate the risk factors of CVD-specific mortality and PSCC-specific mortality. We used the Cox proportional hazards regression model to calculate the risk of all-cause mortality. All results were expressed as hazard ratios (HR) and 95% CI. The cumulative incidence function curves of PSCC and CVD mortality were described using parameter estimates and the baseline survivor function from the Fine-Gray model (11).

The R version 3.6.3 was used for all statistical analysis, and a P <.050 was recognized as significant.

## Results

### Patients characteristics

Finally, we enrolled 2668 cases diagnosed with penile squamous cell carcinoma from 2005 to 2016. The mean age at diagnosis was 64.91 (standard deviation =13.72). 2226 (83.4%) patients were white, and 1452 (54.4%) cases were married. There were 1173(44.0%) patients on the pacific coast. The localized stage was the most common (51.8%). There were 2410(90.3%) cases undergoing surgery (**Table1**).

**Table 1:** Description of population-based cohort of men with PSCC.

Characteristic	n	%
**Overall**	2668	100%
**Age at diagnosis, mean (SD)**	64.91(13.72)	
<50 years	391	14.7%
50-59 years	497	18.6%
60-69 years	736	27.6%
≥70 years	1044	39.1%
**Race**		
White	2226	83.4%
Black	261	9.8%
Other^a^	145	5.4%
Unknown	36	1.3%
**Marital status**		
Married	1452	54.4%
SDW	489	18.3%
Single	500	18.7%
Unknown	227	8.5%
**Region**		
East	1115	41.8%
Northern Plains	228	8.5%
Pacific Coast	1173	44.0%
Southwest	152	5.7%
**Year of diagnosis**		
2005-2010	1212	45.4%
2011-2016	1456	54.6%
**Summary stage**		
Localized	1383	51.8%
Regional	789	29.6%
Distant	153	5.7%
Unknown	343	12.9%
**Undergoing surgery**		
No/unknown	258	9.7%
Yes	2410	90.3%
**Cause of death**		
Alive	1576	59.1%
PSCC	463	17.4%
CVD	208	7.8%
Other cancers	189	7.1%
Other cause	232	8.7%

a: American/Indian/Alaska/Native and Asian/Pacific Islander.

SDW, Separated, Divorced, Widowed; SD, standard deviation; PSCC, penile squamous cell carcinoma; CVD, cardiovascular diseases.

### Proportional mortality ratios among PSCC patients

**Figure 1:**
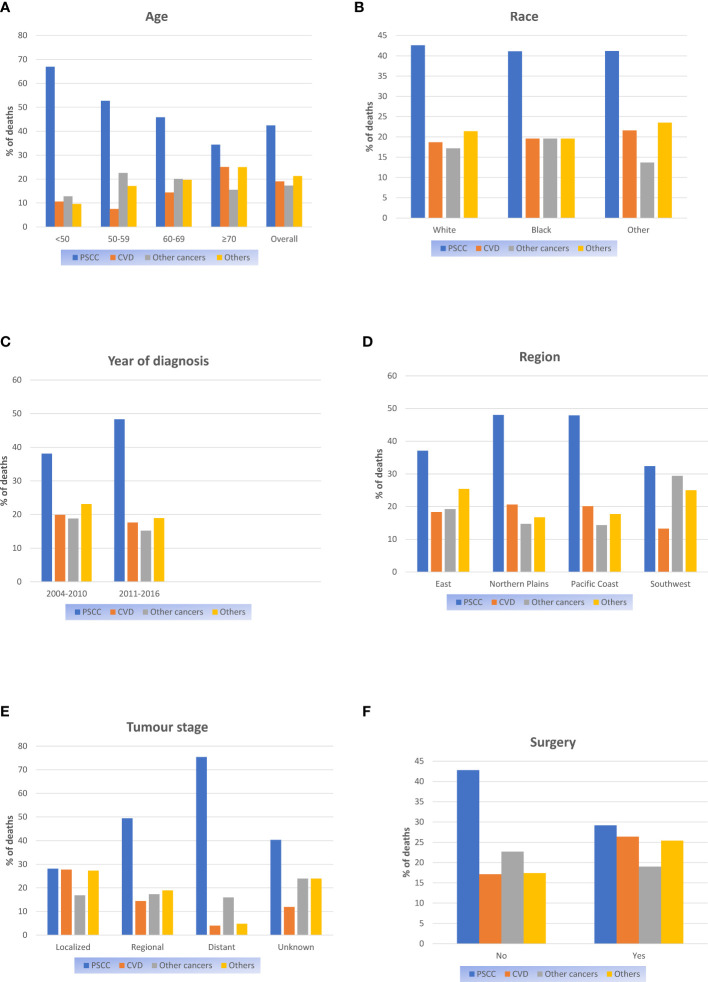
Proportional mortality ratios according to **(A)** Age; **(B)** Race; **(C)** Year of diagnosis; **(D)** Region; **(E)** Tumour stage; **(F)** Surgery.

Proportional mortality ratios of all study populations were as follows: PSCC (42.4%), other causes (21.3%) CVD (19%), and other cancers (17.3%). (**Figure 1A**). In the subgroup analysis, PSCC was still the four most common causes of death among patients in all subgroups. Patients with age over 70 years (25.1%), race of other (21.6%), diagnosed between 2005-2010(19.9%), in the northern plains (20.6%) and with localized stage tumours (27.7%), the PMR of CVD death was relatively higher compared to the other cases in the same subgroup. (**Figure 1**).

### Cardiovascular diseases and all-cause mortality compared to the general population in the USA

The age-specific risk of CVD death and all-cause mortality were compared between patients with PSCC and general men in the USA (**Table 2**). For all study populations, patients with PSCC were observed to have a 3.2(3.1-3.3) times higher risk of cardiovascular death than the general men population, and this value was 6.1(6-6.3) times in terms of all-cause death. Similar results were observed for patients with different ages. Notably, this higher risk of CVD-specific death than the general men population was more significant in PSCC patients with younger age and localized stage (Maximum SMR). In addition, patients receiving surgery had a relatively higher SMR of CVD compared to those without.

**Table 2:** The CVD-specific and all-cause mortality rates of patients with PSCC were standardized by age, surgery and cancer stage relative to the general population of the United States.

	CVD mortality			All-cause mortality		
Category	Observed deaths	Expected deaths	SMR (95%CI)	Observed deaths	Expected deaths	SMR (95%CI)
**Overall**	208	66	3.2 (3.1-3.3)	1092	181	6.1 (6-6.3)
**Age**						
<55	15	2	15 (6.7-23)	149	25	12.1 (7.9-16.3)
55–64	22	5	8.8 (5.4-12.1)	207	26	8.1 (5.5-10.7)
65–74	53	12	4.4 (3.9-5.1)	285	38	6.8 (6.6-7.1)
≥75	118	47	2.5 (2.3-2.7)	451	102	4.5 (4.3-4.7)
**Stage**						
Localized	135	10	13.5 (11.7-15.4)	486	276	1.8 (1.5-2.1)
Regional	60	47	1.3 (0.9-2.3)	417	157	2.7 (2.4-2.9)
Distant	5	9	0.6 (0.3-2.9)	124	31	4.1 (3.8-4.4)
**Undergoing surgery**						
No/Unknown	15	14	1.1 (0.7-2.6)	150	51	2.9 (2.5-3.5)
Yes	193	72	2.7 (2.4-3.2)	942	482	1.9 (1.6-2.3)

PSCC, penile squamous cell carcinoma; CVD, cardiovascular diseases; SMR, standardized mortality ratio; CI, confidence interval.

### Cumulative CVD-specific mortality and Relative risk model

**Figure 2:**
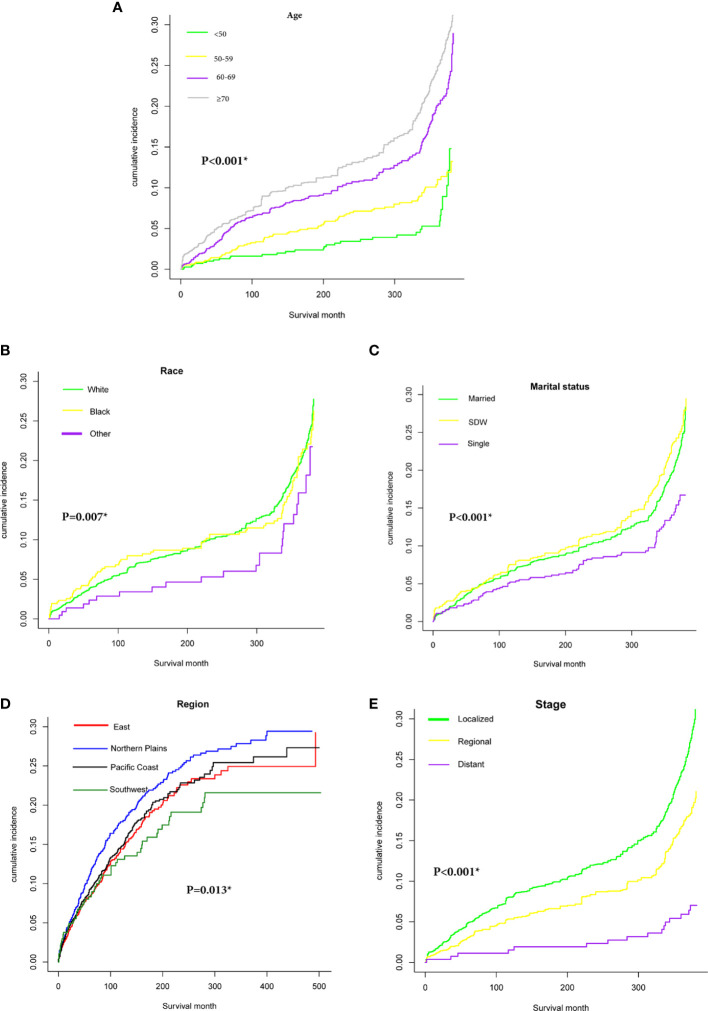
Cumulative incidence function curve of PSCC patients after adjust for multivariate. Cardiovascular diseases (CVD)-specific death: **(A)** age (≤50, 50-59, 60-69, ≥70); **(B)** Race (white, black, other); **(C)** Marital status (Married, SDW, Single); **(D)**: Region (East, Northern Plains, Pacific Coast, Southwest); **(E)** Stage (Localized, Regional, Distant).

**Figure 2** presented cumulative incidence function curves for CVD mortality stratified by population characteristics (We had hidden the lines of ends of PSCC and other causes of death). We observed a relatively higher CVD-specific mortality rate in patients over 70 years and status of SDW compared with other cases in the same cohort (**Figures 2A, C**). Meanwhile, patients diagnosed in the northern plains had a relatively higher incidence of CVD-specific death than other regions (**Figure 2D**). Patients with localized stage showed a remarkably higher cumulative incidence of CVD-specific deaths than those diagnosed with the regional or distant stage (**Figure 2E**).

The Fine–gray model of competitive risk was utilized to explore the associations between prognostic factors and CVD-specific death or PSCC-specific death in PSCC patients (**Table 3**). We found that age was the most significant prognostic factor affecting CVD-specific death, and the risk of CVD-specific death had gradually increased with each 10-year advance in age [from SHR (sub-distribution hazard ratio) = 1.35; 95%CI (1.01- 1.8) in patients aged 50-59 to SHR=3.04; 95%CI (2.36- 3.9) in patients aged over 70]. Compared with those in the region east, patients in the northern plains, pacific coast, or southwest showed a higher risk of CVD-specific death (all SHR>1, P<0.05). Compared with cases diagnosed from 2005 to 2010, patients in 2011-2016 had a relatively lower risk of CVD-specific death (all SHR<1, P<0.001). The risk of CVD-specific death in patients with the regional or distant stage was lower relative to those diagnosed with the localized stage(P<0.001). Married patients presented a relatively lower risk of CVD-specific death than those with SDW and single status.

**Table 3:** All-cause, CVD-related, and PSCC-related mortality of patients by demographic and clinical characteristics at diagnosis.

	All causes		CVD-specific death		PSCC-specific death	
	HR (95%CI)	P-value	SHR (95%CI)	P-value	SHR (95%CI)	P-value
Age						
<50	Ref.		Ref.		Ref.	
50-59	1.17 (0.89- 1.52)	0.24	1.35 (1.01- 1.8)	<0.001^*^	0.89 (0.63-1.25)	0.51
60-69	1.55 (1.22-1.98)	<0.001^*^	1.72 (1.31- 2.24)	<0.001^*^	1.04 (0.75-1.43)	0.81
≥70	3.18 (2.54-4.1)	<0.001^*^	3.04 (2.36- 3.9)	<0.001^*^	1.58 (1.17-2.14)	<0.001^*^
**Race**						
White	Ref.		Ref.		Ref.	
Black	1.18 (0.96-1.45)	0.1	0.91 (0.87-1.37)	0.46	1.1 (0.79-1.5)	0.58
Others	0.77 (0.58-1.04)	0.08	1.18 (0.61-1.17)	0.16	0.73 (0.46-1.15)	0.68
Unknown	0.09 (0.01-0.64)	0.016^*^	0.28 (0.04-1.59)	0.36	0.24 (0.03-1.78)	0.16
**Marital status**						
Married	Ref.		Ref.		Ref.	
SDW	1.33 (1.15-1.55)	<0.001^*^	1.36 (1.15-1.61)	<0.001^*^	1.2 (1.02-1.42)	0.02^*^
Single	1.42 (1.2-1.67)	<0.001^*^	1.43 (1.19-1.73)	<0.001^*^	1.33 (1.04-1.7)	0.022^*^
Unknown	1.11 (0.86-1.42)	0.42	0.88 (0.86-1.48)	0.38	0.836 (0.53-1.31)	0.43
**Region**						
East	Ref.		Ref.		Ref.	
Northern Plains	0.88 (0.71-1.09)	0.24	1.13 (1.1-1.51)	<0.001^*^	1.23 (0.89-1.71)	0.19
Pacific Coast	0.88 (0.77-1.01)	0.075	1.9 (1.71-2.13)	<0.001^*^	1.12 (0.91-1.37)	0.27
Southwest	1.11 (0.86-1.44)	0.43	2.7 (2.04-3.57)	<0.001^*^	0.93 (0.59-1.47)	0.78
**Year of diagnosis**						
2005-2010	Ref.		Ref.		Ref.	
2011-2016	0.99 (0.87-1.13)	0.95	0.57 (0.49-0.66)	<0.001^*^	0.96 (0.79-1.17)	0.74
**Grade**						
Grade I	Ref.		Ref.		Ref.	
Grade II	1.39 (1.18-1.65)	<0.001^*^	0.88 (0.68-1.1)	0.06	2.001 (1.64-2.46)	<0.001^*^
Grade III-IV	1.62 (1.34-1.96)	<0.001^*^	0.98 (0.71-1.09)	0.44	2.12 (1.7-2.66)	<0.001^*^
Unknown	0.96 (0.77-1.21)	0.77	1.08 (0.88-1.32)	0.68	1.33 (1.03-1.71)	0.026^*^
**Stage**						
Localized	Ref.		Ref.		Ref.	
Regional	1.7 (1.48-1.95)	<0.001^*^	0.54 (0.41-0.67)	<0.001^*^	1.93 (1.43-2.59)	<0.001^*^
Distant	5.42 (4.37-6.72)	<0.001^*^	0.41 (0.25-0.69)	<0.001^*^	2.33 (1.69-3.21)	<0.001^*^
Unknown	1.71 (1.29-2.25)	<0.001^*^	0.68 (0.46-0.96)	0.03^*^	1.23 (0.83-1.83)	0.29

SDW, Separated, Divorced, Widowed; SHR, sub-distribution hazard ratio; HR, hazard ratio; CI, confidence interval; Ref, Reference.

*: Statistical significance.

The tumour stage was the most significant prognostic factor associated with PSCC-specific death. Patients with regional or distant stage had a significantly higher risk of PSCC-specific death than those with the localized stage (SHR=1.93, 95%CI: 1.43-2.59, P<0.001 for the regional stage; SHR=2.33, 95%CI: 1.69-3.21, P<0.001 for distant stage). Meanwhile, the status of SDW of single, age, and higher tumour grade were all proved to associate with PSCC-specific death.

## Discussion

This study found that cardiovascular disease was the important cause of death after penile squamous cell carcinomas diagnosis based on a large study population. PSCC patients showed a significantly higher CVD-specific mortality rate than the general men population. We also identified associated factors including age, region, year of diagnosis, stage, and marital status associated with CVD-specific death from the competing risks model.

PSCC was uncommon, especially in developed countries, and it was also an aggressive disease with a relatively high mortality rate (12). Patients with the localized disease were generally effectively treated with topical therapy, surgery, or radiotherapy (2). For patients with metastatic disease, multimodal management was required considering that there was still no strong evidence to confirm the best optimal sequencing of treatments and patient selection (2). Cisplatin-based chemotherapy regimens were commonly used in these patients, but high rates of toxic effects had always been a problem that cannot be ignored (13, 14). In this study, we observed higher CVD-specific mortality in patients undergoing surgery compared with the general population. Although organ preservation technology has been developed for patients with cT0-2 disease in order to maximize the protection of penile tissue and function, the impact of surgery on the quality of life of patients was still profound (15). Penile amputation could lead to the loss of sexual and urinary function. Patients after surgery often experience mental illness, avoidance behavior, health damage, and post-traumatic stress disorder based on questionnaire investigation (16, 17). This effect might be an important cause contributing to the higher CVD-specific mortality. It might be that the survival time of patients with metastatic diseases was too short of supporting this observation, considering that the late effects of cancer treatment might become clinically obvious years or decades after the treatment was completed (18).

Cardiotoxicity was a potential complication in the treatment of various cancers. The new ESMO Clinical Practice Guidelines are based on the multidisciplinary cardio-oncology review of current evidence intended to provide strict standards-based advice on cardiovascular risk prevention, assessment, monitoring, and management during antineoplastic therapy (19). In general, the risk-benefit ratio of drugs needed more consideration in terms of the nature and severity of cancer.

In this study, age was the most significant factor associated with CVD-specific death in PSCC patients (maximal SHR in age over 70years). Meanwhile, we performed a comparison in the risk of CVD-specific death between PSCC patients and the general population and observed a significantly higher risk in PSCC patients. Data derived from childhood cancer proved that the cardiac mortality rate of child cancer survivors was 8.2 times higher than that of the age-matched and gender-matched populations (20). Similarly, the risk of cardiac death was significantly higher in younger patients than in older patients when compared with the general population in our study. This cardiovascular death from cancer or cancer treatment might be more noteworthy in young patients. Another remarkable result was that patients diagnosed with localized stage had a significantly higher risk of CVD-specific death than those diagnosed with regional or distant stage. One explanation for this phenomenon was that patients diagnosed with the localized stage survive significantly longer than those with metastatic diseases (2, 21). Therefore, it seemed no surprise that the probability of cardiovascular events increased in patients with localized stages. Moreover, the “discrepancy” in risk of CVD-specific death was likely explained by the competing death events (PSCC-specific death) incorporated in the Fine-Gray model. Patients with regional or distant stages normally were more likely to die from PSCC but not cardiovascular diseases, which influenced the cumulative incidence of CVD mortality. It was hard to make an explanation for the association between factors (marital status and region) and CVD-specific death. One possible explanation was that some important risk factors for cardiovascular death, such as smoking, alcohol consumption, and high-fat diet, were distributed differently in the study population. Meanwhile, there were great differences in the treatment of PSCC in different regions, and some radiotherapy or chemotherapy techniques might be an important cause of high cardiovascular death in different regions.

Cancer and heart disease are the main causes of morbidity and mortality in industrialized countries (18). With the development of modern treatment strategies, the survival of cancer patients has been significantly improved. However, some studies have pointed out that the overall survival of PSCC patients has not effectively improved since the 1990s in the United States (6). This might be due to the diagnostic delay in the advanced stage and limited nursing and disease management improvements. On the contrary, the overall survival of penile cancer had improved significantly after establishing centralized health care in the U.K (22). This was the result of more appropriate management for penile cancer. In the future, co-morbidities, especially cardiovascular disease, might be an important aspect of improving the survival of PSCC patients, and a more appropriate medical management and nursing system should also be established for those with high-risk cardiovascular diseases.

This study has several limitations to note. First, this study was a retrospective study, and the selection bias caused by itself was inevitable. Then, the SEER program recorded the cause of death from the death certificate information, and this mode tended to overestimate the frequency of cardiovascular events like coronary heart disease (23, 24). In particular, reported data showed that the death certificate accuracy in the SEER database was not relatively satisfactory regarding some cancers. (87% for pancreatic cancer, but there lacked data on PSCC). We expect the impact on the study results was minimal. Furthermore, we used SMR to present the discrepancy in the CVD mortality rates between PSCC patients and the general population. In this case, we ignored PSCC patients in the general male population. Compared with the general population, the low incidence of this disease made our SMR estimation less likely to be biased (2). In addition, due to the limitations of the SEER database, we were unable to obtain more detailed data on PSCC patients like HPV infection, BMI, comorbidity, and smoking behavior, which were all associated with PSCC and cardiovascular diseases.

## Conclusion

Our study mainly reveals that cardiovascular disease was the important cause of death among PSCC patients. PSCC patients showed a significantly higher CVD-specific mortality rate than the general men population. We also identified several associated factors including age, region, year of diagnosis, stage, and marital status associated with CVD-specific death among PSCC patients. Notably, patients diagnosed with localized stage showed a significantly higher cardiovascular disease mortality than those with regional stage or distant stage. Cancer survivorship care is an emerging and necessary component of oncology management. Considering that the survival of patients with PSCC has not improved significantly in recent years, this may prompt the cancer management transition from active cancer treatment to survivorship care, although it is not easy. In the future, more work in educating health care professionals on the components of survivorship care is needed to meet the long-term and late effects cancer patients experience.

## Data availability statement

The original contributions presented in the study are included in the article/Supplementary Material. Further inquiries can be directed to the corresponding authors.

## Ethics statement

Ethical review and approval were not required for the study of human participants in accordance with the local legislation and institutional requirements. Written informed consent from the patients was not required to participate in this study in accordance with the national legislation and the institutional requirements.

## Author contributions

XZ was responsible for data collection and analysis. The manuscript was written by MJ and BF. All authors contributed to the article and approved the submitted version.

## Funding

This study was supported by the National Natural Science Foundation of P.R. China (Grant Nos. 81560419, 81960512, and 81760457) and Jiangxi Provincial “Double Thousand Plan” Fund Project (Grant No. jxsq2019201027).

## Conflict of interest

The authors declare that the research was conducted in the absence of any commercial or financial relationships that could be construed as a potential conflict of interest.

## Publisher’s note

All claims expressed in this article are solely those of the authors and do not necessarily represent those of their affiliated organizations, or those of the publisher, the editors and the reviewers. Any product that may be evaluated in this article, or claim that may be made by its manufacturer, is not guaranteed or endorsed by the publisher.
